# Detecting influenza-associated pulmonary aspergillosis by determination of galactomannan in broncho-alveolar lavage fluid and in serum: should we add (1,3)-beta-D-glucan to improve efficacy

**DOI:** 10.1186/s13054-020-03030-1

**Published:** 2020-06-05

**Authors:** Patrick M. Honore, Leonel Barreto Gutierrez, Luc Kugener, Sebastien Redant, Rachid Attou, Andrea Gallerani, David De Bels

**Affiliations:** ICU Department, Centre Hospitalier Universitaire Brugmann-Brugmann University Hospital, Place Van Gehuchtenplein, 4, 1020 Brussels, Belgium

We read with great interest the recent research letter by Thevissen et al. who reported the findings of their international survey regarding the detection of influenza-associated pulmonary aspergillosis (IAPA), with a focus on the use of galactomannan (GM) in broncho-alveolar lavage (BAL) fluid and serum [1]. They note that greater awareness of IAPA is needed, as are rapid diagnostic tests [1]. We would like to make some comments. Indeed, over the past decades, the patient population having invasive aspergillosis (IA) or IAPA risk factors has expanded significantly, and given that IA/IAPA is associated with high morbidity and mortality, improved diagnostic modalities are required [2]. GM is currently a commonly used method and has a high specificity for IA/IAPA diagnosis, while another test, the (1,3)-beta-D-glucan (BDG) assay, has a high negative predictive value (NPV), making it quite useful to rule out IA/IAPA rather than to confirm it [2, 3]. GM and BDG assays can play an important role in IA/IAPA diagnosis in non-neutropenic patients with underlying respiratory diseases without hematologic malignancy [2]. BDG is the most important and abundant polysaccharide component of the cell wall of most fungi. While incorporated within the fungal cell wall, BDG typically exists as an insoluble structure. In the presence of blood or other body fluids, it transforms into single helix, triple helix (most commonly), or random coil forms and is rendered soluble [4]. The GM assay has been found to be more specific than BDG (97% versus 82%) and BDG more sensitive than GM (81% versus 49%), suggesting that a combination of both tests could strengthen the diagnosis of IA/IAPA [3]. We would like to conclude that GM was found to have high diagnostic specificity, while BDG displayed better sensitivity. Either test used alone carries a certain level of diagnostic limitation. A combination of both assays would improve the diagnostic capacity.

## Data Availability

Not applicable.

## Abbreviations

IAPA: Influenza-associated pulmonary aspergillosis
GM: Galactomannan
BAL: Broncho-alveolar lavage
IA: Invasive aspergillosis
BDG: (1,3)-Beta-D-glucan
